# Early inflammation-driven damage and delayed viral clearance are associated with prolonged biliary perturbation in a Cameroonian cohort of COVID-19 patients

**DOI:** 10.1371/journal.pone.0357503

**Published:** 2026-10-16

**Authors:** Modeste R. Ngamaleu, Hillary Tene, Romeo Djounda, Rachel Minomo, Micheal Besong, Honore Awanakam, Christela Djeukeng, Kevine Tchamda, Reine Seumko’o, Tatiane Feugo, Antonin Wilson Ndjitoyap Ndam, Antoine B. Siafa, Eleonore Ngounoue, Rene Essomba, Charles Kouanfack, Jude Bigoga, Forgu Esemu Livo

**Affiliations:** 1 Department of Biochemistry, University of Yaoundé I, Yaoundé, Cameroon; 2 Centre for Research on Emerging and Re-emerging Diseases, Institute of Medical Research and Medicinal Plant Studies, Ministry of Scientific Research and Innovation, Yaoundé, Cameroon; 3 Catholic University of Central Africa, Higher School of Health Sciences, Yaoundé, Cameroon; 4 Immunology laboratory, The Biotechnology Centre, University of Yaoundé I, Yaoundé, Cameroon; 5 Faculty of Medicine and Biomedical Sciences, University of Yaoundé 1, Yaoundé, Cameroon; 6 University Teaching Hospital Centre of Yaoundé, Yaoundé, Cameroon; 7 Department of Biomedical Sciences, Faculty of Health Sciences, University of Buea, Buea, Cameroon; 8 National Public Health Laboratory, Ministry of Public Health, Yaoundé, Cameroon; Makerere University, UGANDA

## Abstract

Although COVID-19-associated liver abnormalities are documented, the link between delayed viral clearance, IgG seroconversion, and inflammation remains poorly defined, particularly in Sub-Saharan African populations, where baseline liver enzymes differ from Western standards. In 38 SARS-CoV-2-positive individuals monitored at D0, D14, and D30, we quantified liver biomarkers (ALT, AST, BILT, BILD) by spectrophotometry (COBAS), plasma IL-6 by Luminex, viral load (N and ORF1ab genes) by RT-qPCR, and IgG/IgM serology. Analyses were performed in GraphPad Prism v9.0. ALT and AST rose transiently at D14, returning to baseline by D30. IL-6 peaked at D14 and correlated positively with BILT (r = 0.64, p = 0.001) and BILD (r = 0.54, p = 0.006). ORF1ab Ct at D14 correlated negatively with ALT at D0 (r = −0.67, p = 0.004) and D14 (r = −0.71, p = 0.006); N gene Ct at D14 correlated with ALT at D0 (r = −0.61, p = 0.011). Mixed-effects models identified IL-6 as the sole independent predictor of AST (β = 3.04, p = 0.005) and Ct N gene as an independent predictor of direct bilirubin (β = −0.003, p = 0.025), supporting a dual-pathway model of COVID-19-associated cholestasis. Paradoxically, IgG seropositivity independently predicted higher direct bilirubin (β = 0.06, p = 0.030) but did not mark biliary normalisation. Elevated ALT and BILT at D0 were associated with detectable viral RNA beyond D14 (P = 0.03). Participants aged >30 years drove rising BILD, with significant increases at D14 (P = 0.03) and D30 (P = 0.04) versus D0. COVID-19 liver injury follows a biphasic course: D14 alterations are inflammation-driven (IL-6), while D30 abnormalities track with delayed viral RNA clearance independent of IgG seroconversion. Extended hepatic monitoring is warranted, especially in older patients with delayed viral clearance in resource-limited settings.

## Introduction

Since its emergence in Wuhan, China, in late 2019, coronavirus disease 2019 (COVID-19), caused by severe acute respiratory syndrome coronavirus 2 (SARS-CoV-2), has rapidly evolved into a global pandemic [1]. Although SARS-CoV-2 primarily targets the respiratory system, accumulating evidence has firmly established COVID-19 as a systemic disease with multi-organ involvement. Beyond the lungs, SARS-CoV-2 affects the cardiovascular, renal, neurological, and gastrointestinal systems, including the liver [1,2]. Liver dysfunction is a frequent clinical manifestation of COVID-19, reported in approximately 14% to 53% of patients depending on disease severity and study populations [3,4]. Hepatic involvement is most commonly characterised by elevations in alanine aminotransferase (ALT) and aspartate aminotransferase (AST), as well as alterations in total and conjugated bilirubin levels, reflecting hepatocellular injury and cholestatic disturbances [5].

The mechanisms underlying SARS-CoV-2-associated liver injury are complex and multifactorial. Direct viral infection of hepatic tissue has been demonstrated, supported by high expression of angiotensin-converting enzyme 2 (ACE2) and transmembrane protease serine 2 (TMPRSS2) on hepatocytes and cholangiocytes [1,6]. Viral entry through these receptors may induce direct cytopathic effects, including hepatocellular apoptosis and necrosis, thereby leading to elevated transaminase [6]. In parallel, severe COVID-19 is frequently accompanied by a dysregulated immune response, characterised by excessive production of pro-inflammatory cytokines, commonly referred to as a “cytokine storm” [7]. Interleukin-6 (IL-6), a central driver of systemic inflammation and disease severity [8,9]. Critically, elevated IL-6 levels have been strongly correlated with increased ALT and AST, directly linking inflammation to liver injury during acute infection [10,11]. Additional contributing factors include hypoxia secondary to respiratory failure, COVID-19-associated coagulopathy resulting in microvascular thrombosis within the hepatic circulation, and drug-induced liver injury related to antivirals, corticosteroids, and other medications extensively metabolised by the liver [12].

Beyond the acute phase, a growing body of evidence highlights persistent hepatic abnormalities in patients recovering from COVID-19. Recent systematic reviews and meta-analyses have documented that liver enzyme elevations may persist or recur well beyond the acute infection period, suggesting ongoing hepatic involvement as part of the post-acute sequelae of SARS-CoV-2 infection (PASC), commonly referred to as long COVID-19 [13–15]. The mechanisms underlying post-acute liver injury remain incompletely understood, but likely involve a combination of residual direct viral cytotoxicity, sustained local immune activation, and endothelial dysfunction [15]. Of particular interest is the phenomenon of viral persistence, defined as detectable SARS-CoV-2 RNA beyond the initial acute phase, which has been proposed as a key driver of delayed tissue recovery across multiple organ systems [15,16]. However, the specific contribution of prolonged viral shedding to the incomplete normalisation of hepatic biomarkers, independently of systemic inflammation, has not been prospectively characterised.

Recovery from COVID-19 is generally associated with seroconversion, characterised by the detectable appearance of anti–SARS-CoV-2 IgG antibodies, often interpreted as a surrogate marker of immune protection and disease resolution [17]. However, increasing evidence suggests that seroconversion does not necessarily coincide with complete biological recovery. The qualitative aspects of the humoral response, including antibody avidity, neutralising capacity, and Fc-mediated effector functions, appear to be more determinant than antibody detection alone [18,19]. The relationship between IgG seroconversion, viral clearance, and the normalisation of liver biomarkers therefore remains to be clarified, particularly in longitudinal studies conducted over the post-acute phase.

It is increasingly recognised that biological reference intervals for liver enzymes vary significantly across different geographical and ethnic populations. In sub-Saharan African populations, particularly in Cameroon, baseline levels of transaminases (ALT and AST) have been reported to exceed those established for Western cohorts, likely due to a combination of genetic factors, dietary patterns, endemic co-infections, and environmental exposures [20,21]. These differences have important clinical implications, as standard diagnostic thresholds derived from European or North American cohorts may misclassify the severity of hepatic involvement in African patients. Despite this recognised biological heterogeneity, longitudinal data characterising the post-acute immunovirological and biochemical evolution of COVID-19 in sub-Saharan African populations remain scarce. This gap is particularly consequential in a context characterised by a high prevalence of comorbidities, including HIV infection, malaria, as well as frequent use of traditional herbal medicines with potential hepatotoxic effects [22]. The intersection of these local epidemiological features with SARS-CoV-2-induced hepatic perturbations a largely unexplored research frontier [5,23,24].

Against this background, the present prospective longitudinal study, conducted in Yaoundé, Cameroon, aimed to characterise the temporal dynamics of liver dysfunction in SARS-CoV-2-infected patients over 30 days following diagnosis, in relation to inflammatory responses, viral persistence, and IgG serostatus. By directly examining the interplay among these biological axes in an understudied African cohort, this study seeks to fill a critical gap in understanding the drivers of post-acute hepatic injury beyond the clinical frameworks derived from high-income-country settings.

## Materials and methods

### Study design and participants

This longitudinal observational study enrolled 38 SARS-CoV-2–confirmed participants of both sexes and across all age groups, recruited between January and April 2021 during the second epidemic wave of COVID-19 in Cameroon. Participants were recruited at the Centre for Research on Emerging and Re-emerging Diseases (CREMER), Yaoundé, Cameroon. SARS-CoV-2 infection was confirmed by reverse transcription quantitative polymerase chain reaction (RT-qPCR). All participants provided written informed consent prior to enrolment. Consent from a parent or legal guardian was obtained for participants under 21 years of age. This study was conducted in accordance with the Declaration of Helsinki and approved by the National Ethics Committee of Cameroon (2020/05/1218/CE/CNERSH/SP) with specific clearance from the Institutional Review Board of Human Health (No. 2021/020407/CEIRSH/ESS/MIM). The data were accessed for research purposes on 20 January 2022

Participants were followed longitudinally at three pre-specified time points: day 0 (D0, day of diagnosis), day 14 (D14), and day 30 (D30). Participants were classified as symptomatic or asymptomatic at enrolment. Symptomatic status was defined as the presence of at least three WHO-defined COVID-19 symptoms (fever, cough, dyspnoea, fatigue, myalgia, headache, sore throat, anosmia, or ageusia) in conjunction with a positive RT-qPCR result. The symptom counts in Table 1 therefore reflect individual symptom frequencies among symptomatic participants, not the number of participants who meet the case definition. Asymptomatic status was defined as RT-qPCR positivity in the complete absence of clinical symptoms at enrolment and during follow-up. Participants with confirmed hepatitis B or hepatitis C co-infection were excluded to avoid confounding effects on liver function biomarkers. The detailed recruitment procedures and clinical characterisation have been previously described [25].

**Table 1:** Demographic and clinical characteristics of the population.

Characteristics	Total	Asymptomatic	Symptomatic	P-value
N (%)	38	21(55.26)	17(44.74)	
Sex (%)				
Female	19(50)	11(57.90)	8(42.1)	0.4
Male	19(50)	10(52.63)	9(47.37)	
Age (years) (%)				
< 30	16(42.10)	8(50)	8(50)	0.1
> 30	22(57.9)	13(59.1)	9(40.9)	
Symptoms				
Sore throat (%)			2 (11.76)	
Cough (%)			6 (35.3)	
Wheezing (%)			3 (17.6)	
Dizziness (%)			1 (5.88)	
Headache (%)			2 (11.76)	
Myalgia (%)			1 (5.88)	
Asthenia (%)			1 (5.88)	
Missing (%)			1 (5.94)	

The following potential hepatic confounders were not formally assessed at enrolment due to the operational constraints of the study protocol, which was designed primarily as a virological and immunological cohort study: non-alcohol consumption, use of traditional herbal medicines, antipyretic medication (particularly paracetamol), and corticosteroid exposure. This represents a recognised limitation of the study and is discussed further in the Limitations section.

### Biochemical measurements

Serum levels of ALT(UI/L), AST(UI/L), BILT (mg/dL), and BILD (mg/dL) were quantified using the COBAS c111 automated biochemical analyser (Roche Diagnostics, Basel, Switzerland), according to the manufacturer’s instructions, using spectrophotometric quantification after analyte-specific enzymatic reactions. Laboratory reference ranges used for clinical interpretation were as follows: ALT 10–50.0 U/L, AST 10–50 U/L, total bilirubin 0.146–1.23 mg/dL, direct bilirubin <0,3 mg/dL as defined by the COBAS c111 manufacturer. The analyte concentration of each sample is automatically calculated using the conversion factor: µkat/L = IU/L × 0.0167. All biochemical analyses were performed in single analytical runs per sample, consistent with standard clinical laboratory practice. Laboratory personnel were not blinded to participant identity; however, all measurements were conducted in strict accordance with standardised protocols and manufacturer instructions to minimise operator-dependent variability.

### Serological IgG/IgM testing for SARS-CoV-2 antibodies

Anti-SARS-CoV-2 IgG and IgM antibodies were detected using the Abbott Panbio™ COVID-19 IgG/IgM Rapid Test Device (Abbott Diagnostics, USA), a qualitative immunochromatographic lateral flow assay validated for use with whole blood, serum, and plasma samples [18]. Serological testing was performed at each of the three follow-up visits (D0, D14, D30). Results were recorded as positive or negative for each antibody class. This assay provides qualitative detection only and does not yield quantitative antibody titres, antibody avidity measurements, or functional neutralisation capacity. Accordingly, all conclusions regarding IgG status in this manuscript refer specifically to qualitative seropositivity and do not reflect the full spectrum of the humoral immune response.

### Measurement of interleukin-6 (IL-6)

At each follow-up visit, 10 mL of venous blood was collected into EDTA tubes and centrifuged using an Eppendorf 5804 centrifuge to obtain plasma. Plasma aliquots were stored at −80 °C until batch analysis. IL-6 concentrations were quantified using a multiplex bead-based Luminex assay, as previously described [25].

### Detection of SARS-CoV-2 RNA and viral persistence

Nasopharyngeal swab samples were collected at D0, D14, and D30 and preserved in viral transport medium. SARS-CoV-2 RNA detection targeting the N and ORF1ab genes was performed by RT-qPCR following protocols previously reported [25]. Cycle threshold (Ct) values were recorded as a proxy for viral load. Ct values are inversely proportional to viral RNA quantity: a lower Ct value indicates that fewer amplification cycles were required to reach the detection threshold, reflecting a higher quantity of viral RNA. Accordingly, a negative correlation between Ct values and a liver biomarker indicates that higher viral RNA abundance is associated with higher biomarker levels. The positivity threshold was set at Ct ≤ 40 in accordance with the WHO interim guidance for SARS-CoV-2 RT-qPCR testing [26]. Viral persistence was defined as detectable SARS-CoV-2 RNA at D14 (Ct ≤ 40 for either N or ORF1ab gene). RT-qPCR positivity reflects the presence of viral RNA and does not confirm viable or replication-competent virus. No viral culture or subgenomic RNA quantification was performed in this study. The term “viral persistence” in this manuscript therefore refers exclusively to delayed RNA clearance, and all related conclusions should be interpreted as associations with delayed virological clearance rather than evidence of ongoing active viral replication.

### Statistical analysis

Data from the 38 participants were entered and curated using Microsoft Excel 2019 and analysed with GraphPad Prism version 9.0 (GraphPad Software, USA) and R version 4.5.1 (R Foundation for Statistical Computing). The age threshold of 30 years was not selected a priori on biological grounds but was determined by the demographic distribution of our cohort, in which the median age was approximately 30 years, making this the natural split point for two balanced subgroups. The normality of all quantitative variables (ALT, AST, BILT, BILD, IL-6 concentrations, and Ct values for N and ORF1ab genes) was assessed using the Shapiro–Wilk test. As most variables did not follow a normal distribution, non-parametric statistical methods were employed throughout. Longitudinal changes across the three time points (D0, D14, and D30) were analysed using the Friedman test, with pairwise post-hoc comparisons performed using the Wilcoxon signed-rank test. Comparisons between two independent groups (IgG-positive vs IgG-negative participants; viral persistence vs no viral persistence) were conducted using the Mann–Whitney U test; Comparisons involving more than two groups (age categories and symptom status) were evaluated using the Kruskal–Wallis test, followed by Dunn’s post-hoc multiple comparison test. Associations between IL-6 levels, viral load indicators (Ct values for N and ORF1ab genes), and liver biomarkers were assessed with Spearman’s rank correlation coefficient. To disentangle the independent contributions of age, sex, symptom status, IL-6, viral RNA abundance (Ct N gene), IgG serostatus, and time point to longitudinal liver biomarker levels, four linear mixed-effects (LME) models were constructed with liver biomarkers as outcomes. All four models included participant as a random effect to account for within-subject correlation across repeated measurements, with random intercepts estimated per individual (τ₀₀). Fixed effects were estimated as unstandardised β coefficients with 95% confidence intervals (CI). Models were fitted to 34 observations from 26 participants with complete data at least two time points. Marginal R² (variance explained by fixed effects alone) and conditional R² (variance explained by both fixed and random effects) are reported as measures of model fit. Given the exploratory nature of these analyses, all results are considered hypothesis-generating. This study was not powered a priori to detect specific effect sizes in the subgroup and correlation analyses reported. All subgroup analyses (by sex, age, symptom status, IgG serostatus, and viral RNA clearance status) are therefore exploratory and hypothesis-generating. Correlation matrices were produced to visualise these relationships. A two-sided p-value < 0.05 was regarded as statistically significant. All relevant data are within the paper and its Supporting Information file (S1 File).

## Results

### Participants characteristics

Of 38 participants enrolled at baseline (Day 0), 30 had liver biomarkers assessed, and 27 had IL-6 measured. At Day 14, liver biomarkers were available for 27 participants (3 lost to follow-up) and IL-6 for 25 (2 lost to follow-up). At Day 30, liver biomarkers were available for 25 participants (2 lost to follow-up) and IL-6 for 22 (3 lost to follow-up). Excluded at Day 0: insufficient serum volume (n = 8); IL-6 below detection limit (n = 11). All analyses are based on complete-case data at each time point. No imputation was performed (Fig 1).

**Fig 1:**
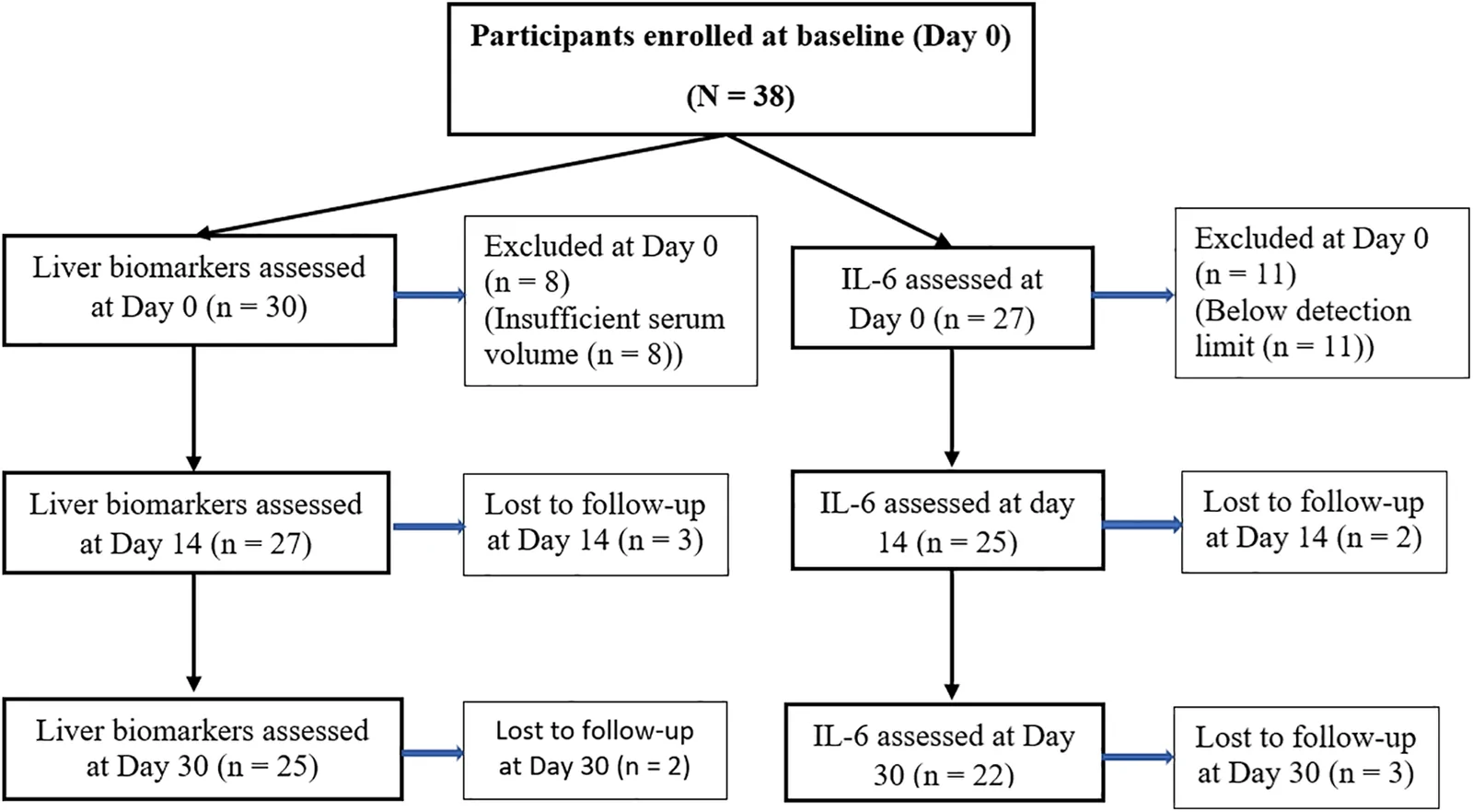
Participant flow diagram from recruitment to follow-up over 30 days.

### Baseline demographic, clinical, and serological characteristics

A total of 38 participants were included in the study, comprising 17 symptomatic (44.7%) and 21 asymptomatic (55.3%) individuals. Men and women were equally represented overall (50% each); however, women predominated in the asymptomatic group (57.9%). The cohort’s mean age was 34.6 years (SD 13.98; IQR 21.25), with 57.9% of participants aged >30 years. Asymptomatic individuals were older than symptomatic patients. The most frequently reported symptoms were cough, followed by respiratory distress, malaise, and sore throat. Baseline demographic and clinical characteristics are summarised in Table 1. The comparison table of sociodemographic data, longitudinal clinical data, and biomarkers after Benjamini-Hochberg adjustment can be found in S1 Table.

At baseline (D0), seropositivity for SARS-CoV-2 antibodies was low. Among symptomatic patients, IgM and IgG were detected in 11.8% and 9.1% of patients, respectively, whereas IgM seroprevalence among asymptomatic individuals was 4.8%. By D30, IgM seropositivity remained higher in symptomatic patients (17.6%) than in asymptomatic individuals (0%), whereas IgG seroprevalence was similar between the two groups (Table 2).

**Table 2:** Evolution of seroprevalence of anti-SARS-CoV-2 IgG/IgM antibodies.

		Overall	Positive Cases		Negative Cases		Missing Data		
Antibody	Timepoint	Overall Positive, n (%)	Symptomatic Pos, n (%)	Asymptomatic Pos, n (%)	Symptomatic Neg, n (%)	Asymptomatic Neg, n (%)	Symptomatic Miss, n (%)	Asymptomatic Miss, n (%)	p-value
IgM	Day 0	3 (7.9%)	2 (11.8%)	1 (4.8%)	15 (88.2%)	19 (90.5%)	0 (0.0%)	1 (4.8%)	0.584
	Day 14	3 (7.9%)	1 (5.9%)	2 (9.5%)	14 (82.4%)	13 (61.9%)	2 (11.8%)	6 (28.6%)	1.000
	Day 30	3 (7.9%)	3 (17.6%)	0 (0.0%)	12 (70.6%)	14 (66.7%)	2 (11.8%)	7 (33.3%)	0.224
IgG	Day 0	3 (7.9%)	1 (5.9%)	2 (9.5%)	16 (94.1%)	18 (85.7%)	0 (0.0%)	1 (4.8%)	1.000
	Day 14	17 (44.7%)	9 (52.9%)	8 (38.1%)	6 (35.3%)	7 (33.3%)	2 (11.8%)	6 (28.6%)	1.000
	Day 30	18 (47.4%)	9 (52.9%)	9 (42.9%)	6 (35.3%)	5 (23.8%)	2 (11.8%)	7 (33.3%)	1.000

Liver biomarkers (ALT, AST, BILT, and BILD) were monitored at three time points (D0, D14, D30) in 38 subjects. Overall, biochemical markers increased from D0 to D30 (see Fig 2a), with a notable rise in direct bilirubin (BILD) at D30 compared with D0 (P = 0.02) across all participants (n = 38). Clinical profile analysis indicates that transaminases (ALT/AST) peak at D14 before stabilising, while bilirubin levels (BILT/BILD) gradually increase, particularly in symptomatic individuals (Fig 2b). These trends are influenced by sex and age (Figs 2c and 2d). Despite equal numbers (15 men and 15 women), men show greater variability in transaminase levels at D14. Participants over 30 years old (n = 17/30) primarily drive the upward trend in BILD, with significant increases at D14 (P = 0.03) and D30 (P = 0.04) compared with baseline (D0). No significant changes were observed in those under 30 (n = 13/30).

**Fig 2:**
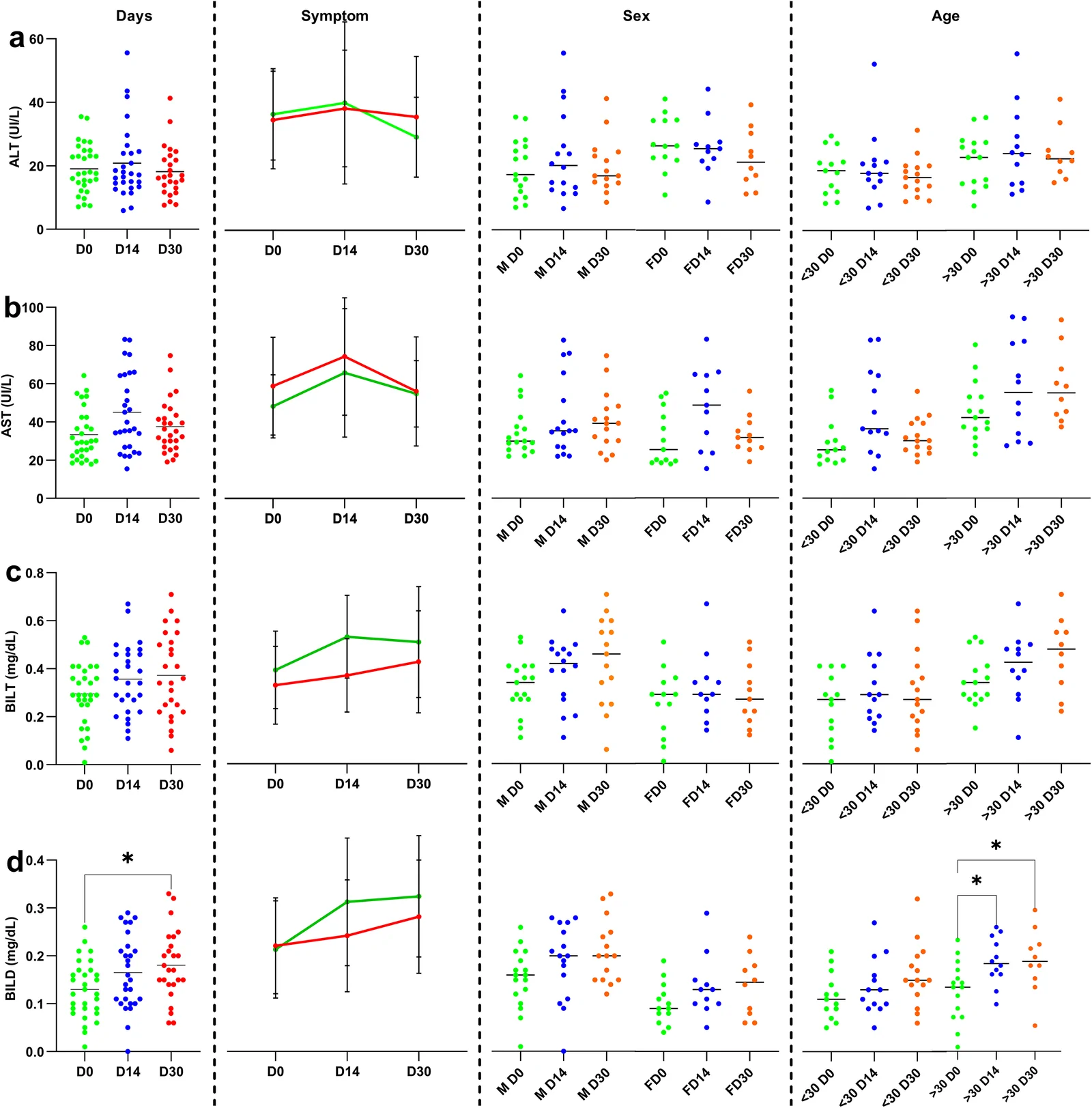
BILD levels increased with times, and was exacerbated by age. Serum levels of (a) ALT (IU/L), (b) AST (IU/L), (c) BILT (mg/dL), and (d) BILD (mg/dL) were recorded on days 0, 14, and 30 for the full cohort (n = 30/38). The data are segmented by day (follow-up visits), clinical status (Symptoms: red line = symptomatic; green line = asymptomatic), gender (Sex: M = males, n = 15/38; F = females, n = 15/38), and age group (Age column: < 30 years, n = 13/38; > 30 years, n = 17/38). Horizontal bars represent mean values, and asterisks (*) mark statistically significant differences (P < 0.05).

The IgG serostatus was assessed alongside liver biomarkers on days 0, 14, and 30 (see Fig 3). At baseline (D0), there were no significant differences between the IgG+ (n = 17/30) and IgG- (n = 13/30) groups across any parameters. Throughout the follow-up period, ALT (Fig 3a) and AST (Fig 3b) levels showed similar distributions in both groups. However, in IgG+ individuals, AST was significantly higher on day 14 (43.80 IU/L, n = 14/17) than on day 30 (32.90 IU/L, n = 15/17). Regarding biliary function, BILT (Fig 3c) and BILD (Fig 3d) showed a gradual increase up to day 30 in both subgroups. As expected, the number of seropositive individuals increased over time, from 14/17 on day 14–15/17 by day 30. However, this seroconversion did not lead to a decrease in biliary markers. Specifically, in the IgG+ group, the median BILT remained stable between day 14 (0.37 mg/dL) and day 30 (0.36 mg/dL). Similarly, the median BILD in IgG+ patients increased slightly, from 0.16 mg/dL on day 14 to 0.18 mg/dL on day 30.

**Fig 3:**
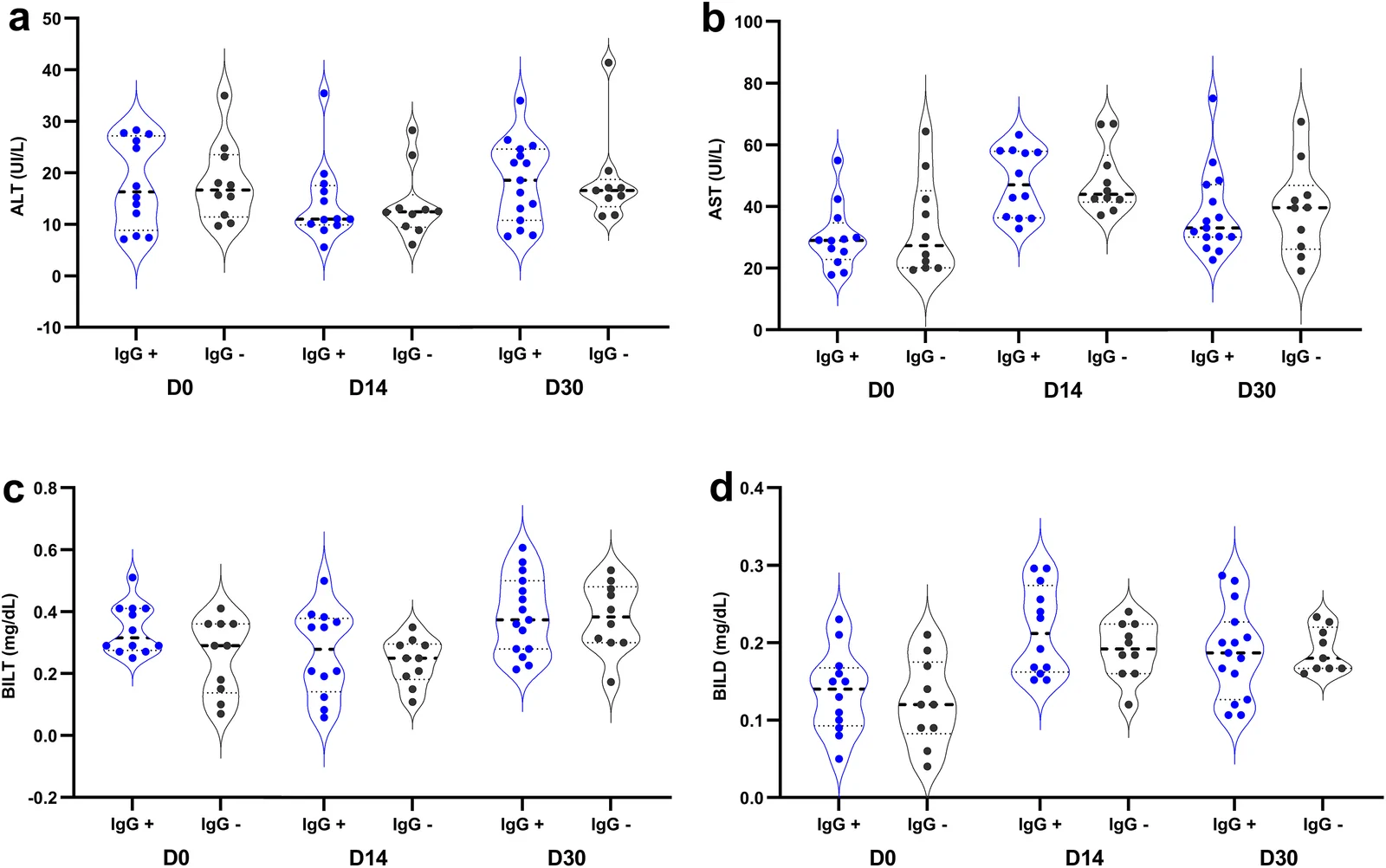
Liver biomarker levels were not significantly affected by COVID IgG serological status. Serum levels of (a) ALT (IU/L), (b) AST (IU/L), (c) BILT (mg/dL), and (d) BILD (mg/dL) are shown as violin plots with individual data points. Measurements were taken at three time points: D0, D14, and D30. The dataset comprises subjects labelled as IgG+ (blue, n = 17/30) and IgG- (black, n = 13/30). Dotted lines in the plots represent the median and quartiles.

A follow-up analysis of interleukin-6 (IL-6) levels across demographic and clinical profiles at various time points (see Fig 4). At inclusion (D0), male subjects (n = 15/27) had significantly higher IL-6 levels than female subjects (n = 12/27; P = 0.04), although this difference decreased at D14 and D30 (see Fig 4a). Symptomatic subjects (n = 10/27) had higher IL-6 levels than asymptomatic subjects (n = 17/27) at D0 (P = 0.01), but by D30, the trend reversed, with higher levels in asymptomatic subjects (P = 0.04; Fig 4b). Individuals over 30 years old (n = 14/24) showed significantly higher IL-6 levels than those under 30 (n = 10/24, P = 0.01), a pattern that remained at D30 (Fig 4e). No significant differences were found regarding IgG status (Fig 4c) or viral load persistence (Fig 4d). Overall, IL-6 levels declined from D0 to D30 across the entire cohort (see Fig 4f).

**Fig 4:**
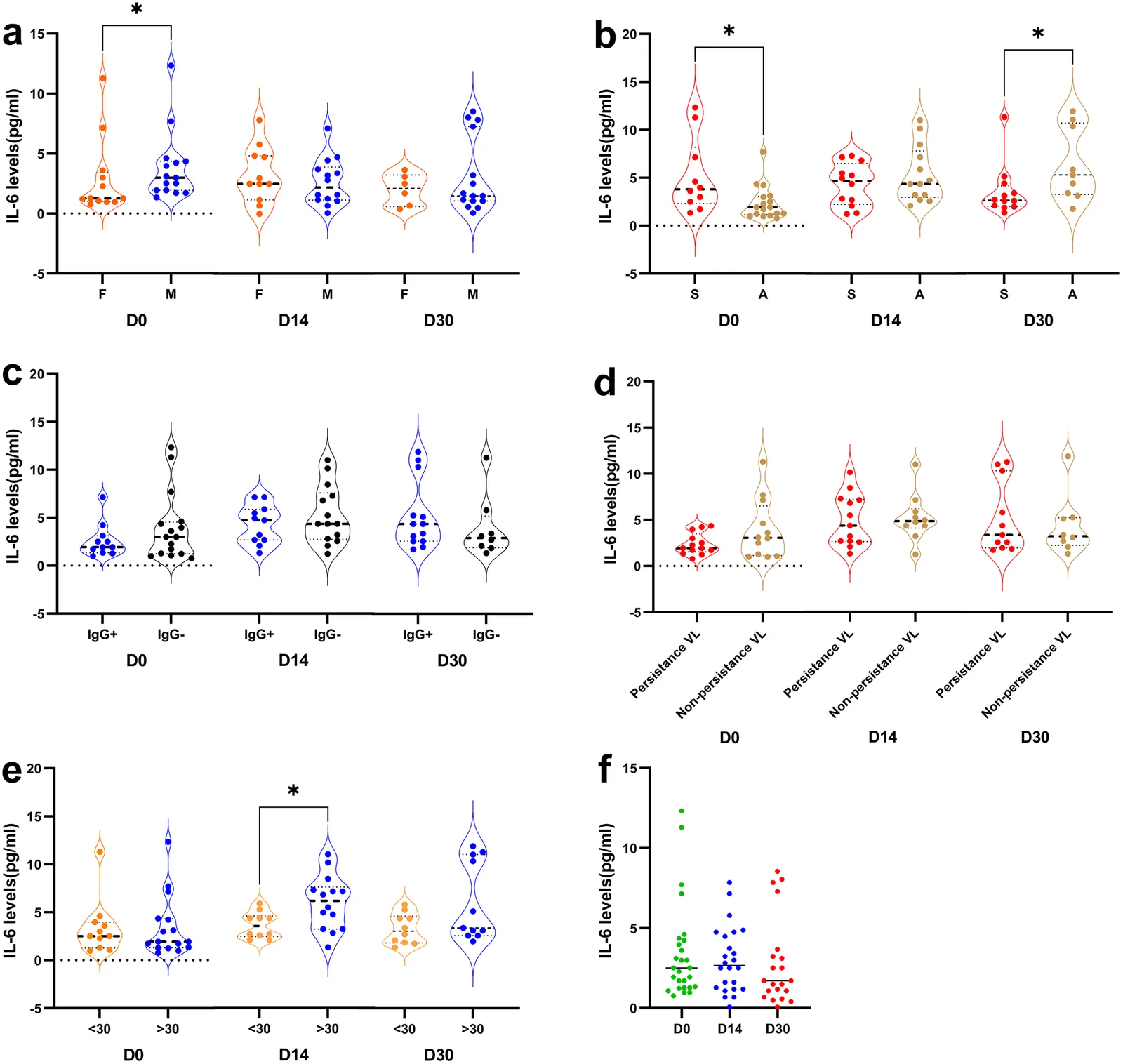
IL-6 levels (pg/ml) were transiently affected by sex, symptoms and age. Measurements were taken at specific time points: D0 (n = 27), D14 (n = 24), and D30 (n = 21). The graphs display IL-6 concentrations by (a) gender (M, F), (b) clinical status (S = symptomatic, A = asymptomatic), (c) IgG serostatus (IgG + , IgG-), (d) viral load persistence, (e) age group (<30 years; > 30 years), and the (f) entire cohort (n = 38). Data are represented as violin plots with individual data points included. Dotted lines mark the median and quartiles. Significant differences (P < 0.05) are indicated with asterisks (*).

RT-qPCR-based viral load analysis, expressed as cycle thresholds (Ct), was conducted on days 0, 14, and 30 for the N (Fig 5a) and ORF1ab (Fig 4b) genes. Fig 5 shows the distribution of Ct values, stratified by gender, age group, and clinical status. The data are shown as violin plots with individual points, medians, and quartiles (dotted lines). There was a gradual decline in the number of subjects with a detectable viral load from D0 to D30, with persistence limited to a few individuals by the end of the follow-up period, regardless of gender, age, or symptomatology.

**Fig 5:**
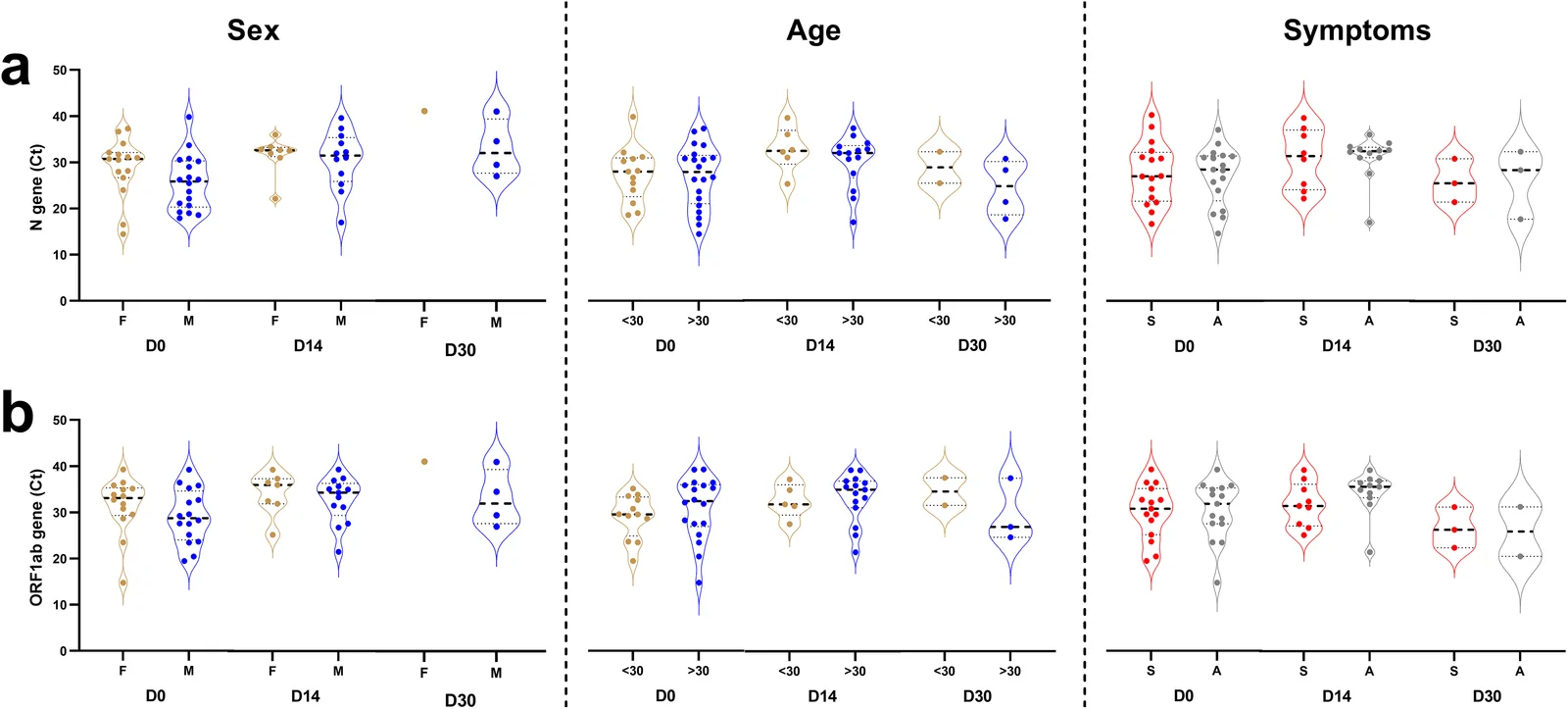
Viral load dynamics (Ct values) was not affected by sex, age, and symptoms for 30 days. Threshold cycles (Ct) for the (a) N gene and the (b) ORF1ab gene were measured by RT-qPCR at D0, D14, and D30. The data are shown as violin plots, with individual points grouped by gender (F; M), age category (<30 years; > 30 years), and clinical status (S = symptomatic; A = asymptomatic). Thick dotted lines mark the medians, while thin dotted lines denote the quartiles.

On day 0 (D0), individuals with a persistent viral load had significantly higher ALT (Fig 6a) and BILT (Fig 6c) levels than those without a delayed viral load (P = 0.03). By D14, a highly significant difference in AST was observed (Fig 6b), with lower levels in participants with delayed viral load than in those without (P = 0.004). No statistical difference was observed for BILD (Fig 6d), although the medians were slightly higher in the delayed group at both D0 and D14.

**Fig 6:**
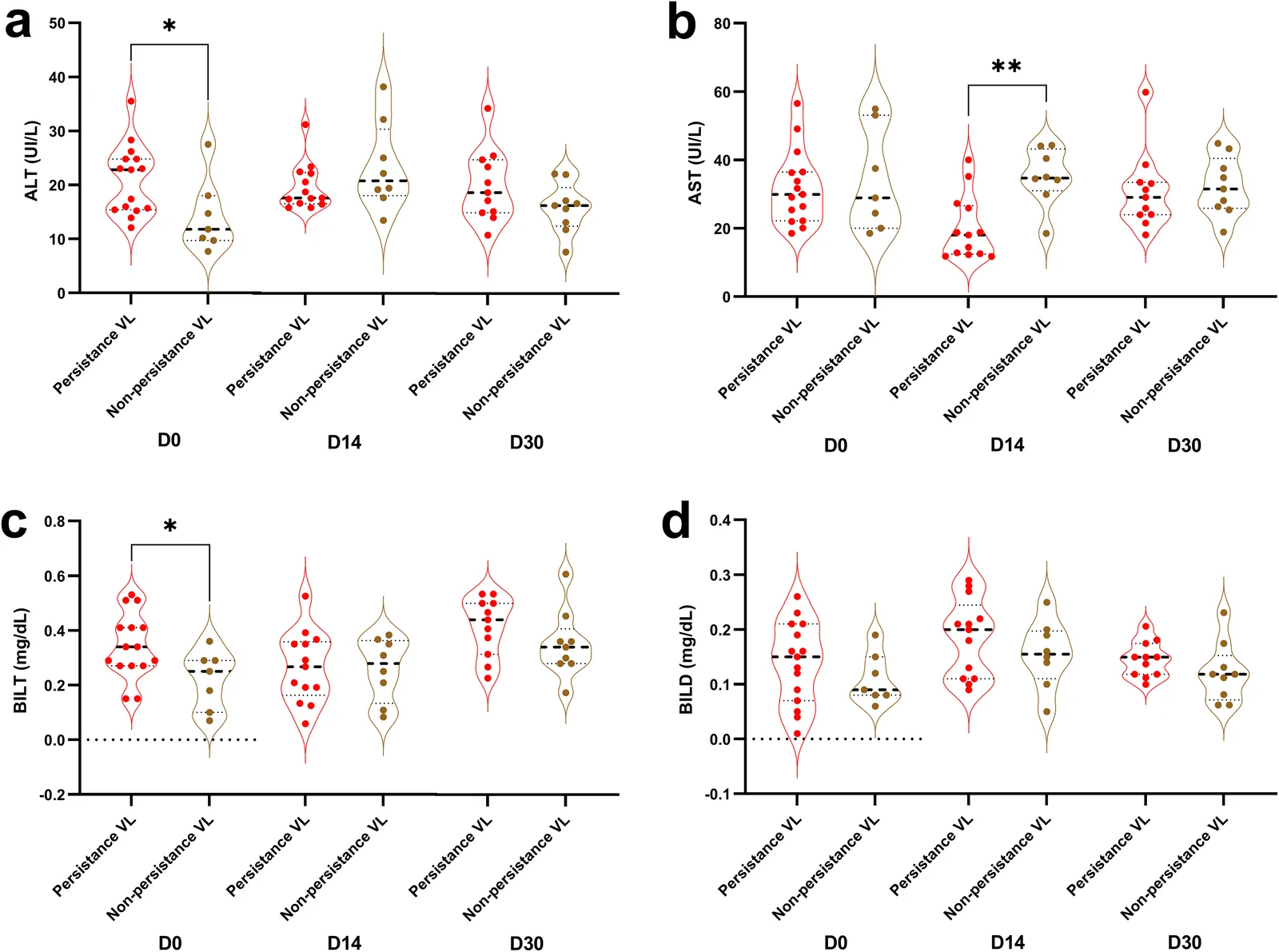
Elevated ALT and BILT levels during diagnosis are predictive of delayed viral clearance. Serum levels of (a) ALT, (b) AST, (c) BILT and (d) BILD are shown for groups with delayed viral load (red) and without (brown) on days 0, 14 and 30. Data are presented as violin plots, showing the distribution of individual points, medians (thick dotted lines) and quartiles (thin dotted lines). Asterisks indicate statistically significant differences: * P < 0.05 and ** P < 0.01.

Multivariable linear mixed-effects models were used to analyse ALT, AST, BILT, and BILD across three time points (34 observations, 26 subjects). IL-6 was the sole significant independent predictor of AST (β = 3.04, 95% CI: 1.03–5.05, p = 0.005), indicating systemic inflammation independently contributes to hepatocellular injury after adjusting for all covariates. For BILD, two predictors were significant: Ct N gene (β = −0.003, 95% CI: −0.006 to −0.000, p = 0.025), suggesting higher viral RNA levels are linked to increased conjugated bilirubin retention, and IgG seropositivity (β = 0.06, 95% CI: 0.01–0.11, p = 0.030), with seropositive participants showing higher BILD despite adjustments for viral load and IL-6. Trends toward association between Ct N gene and ALT (β = −0.35, p = 0.111) and BILT (β = −0.01, p = 0.082) were noted but not significant after full adjustment. High conditional R² values for ALT (0.798) and BILT (0.853), compared with low marginal R² values (0.190 and 0.170), suggest that most long-term variance in these biomarkers is attributable to stable individual differences captured by random intercepts rather than to the measured fixed predictors (Fig 7 and Table 3).

**Table 3:** Mixed-effects multivariable regression models for liver biomarker outcomes across three time points (D0, D14, D30). To disentangle the independent contributions of age, sex, symptom status, IL-6, viral RNA abundance, IgG serostatus, and time point to longitudinal liver biomarker levels, four linear mixed-effects (LME) models were constructed using liver biomarkers as outcomes.

*Predictors*	ALT (UI/L)			AST (UI/L)			BILT (mg/dL)			BILD (mg/dL)		
	*Estimates*	*95% CI*	*p-value*	*Estimates*	*95% CI*	*p-value*	*Estimates*	*95% CI*	*p-value*	*Estimates*	*95% CI*	*p-value*
(Intercept)	25.04	8.35–41.73	**0.005**	36.81	4.13–69.48	**0.029**	0.42	0.16–0.68	**0.003**	0.15	0.03–0.27	**0.017**
Age	0.05	−0.17–0.27	0.655	0.13	−0.24–0.50	0.482	0.00	−0.00–0.01	0.244	0.00	−0.00–0.00	0.050
Sex [M]	1.15	−5.43–7.74	0.721	−3.73	−15.15–7.70	0.507	0.01	−0.10–0.12	0.886	0.03	−0.01–0.07	0.181
Symptom [Symptomatic]	−2.37	−9.35–4.60	0.489	−4.39	−15.80–7.01	0.434	−0.04	−0.15–0.08	0.501	0.00	−0.04–0.04	0.878
IL6	0.67	−0.44–1.79	0.224	3.04	1.03–5.05	**0.005**	−0.01	−0.03–0.01	0.389	0.00	−0.00–0.01	0.495
Ct Ngene	−0.35	−0.79–0.09	0.111	−0.44	−1.34–0.47	0.328	−0.01	−0.01–0.00	0.082	−0.00	−0.01 – −0.00	**0.025**
IgG [Pos]	0.72	−4.27–5.72	0.768	−4.58	−17.56–8.39	0.473	−0.00	−0.08–0.07	0.916	0.06	0.01–0.11	**0.030**
Timepoint [D14]	0.57	−4.28–5.42	0.811	8.72	−3.38–20.81	0.150	0.02	−0.05–0.10	0.482	0.03	−0.02–0.08	0.195
**Random Effects**												
σ²	14.66			162.94			0.00			0.00		
τ₀₀	44.15 Subject			23.48 Subject			0.01 Subject			0.00 Subject		
N	26 Subject			26 Subject			26 Subject			26 Subject		
Observations	34			34			34			34		
Marginal R²/ Conditional R²	0.190/ 0.798			0.329/ 0.413			0.170/ 0.853			0.476/ NA		

**Fig 7:**
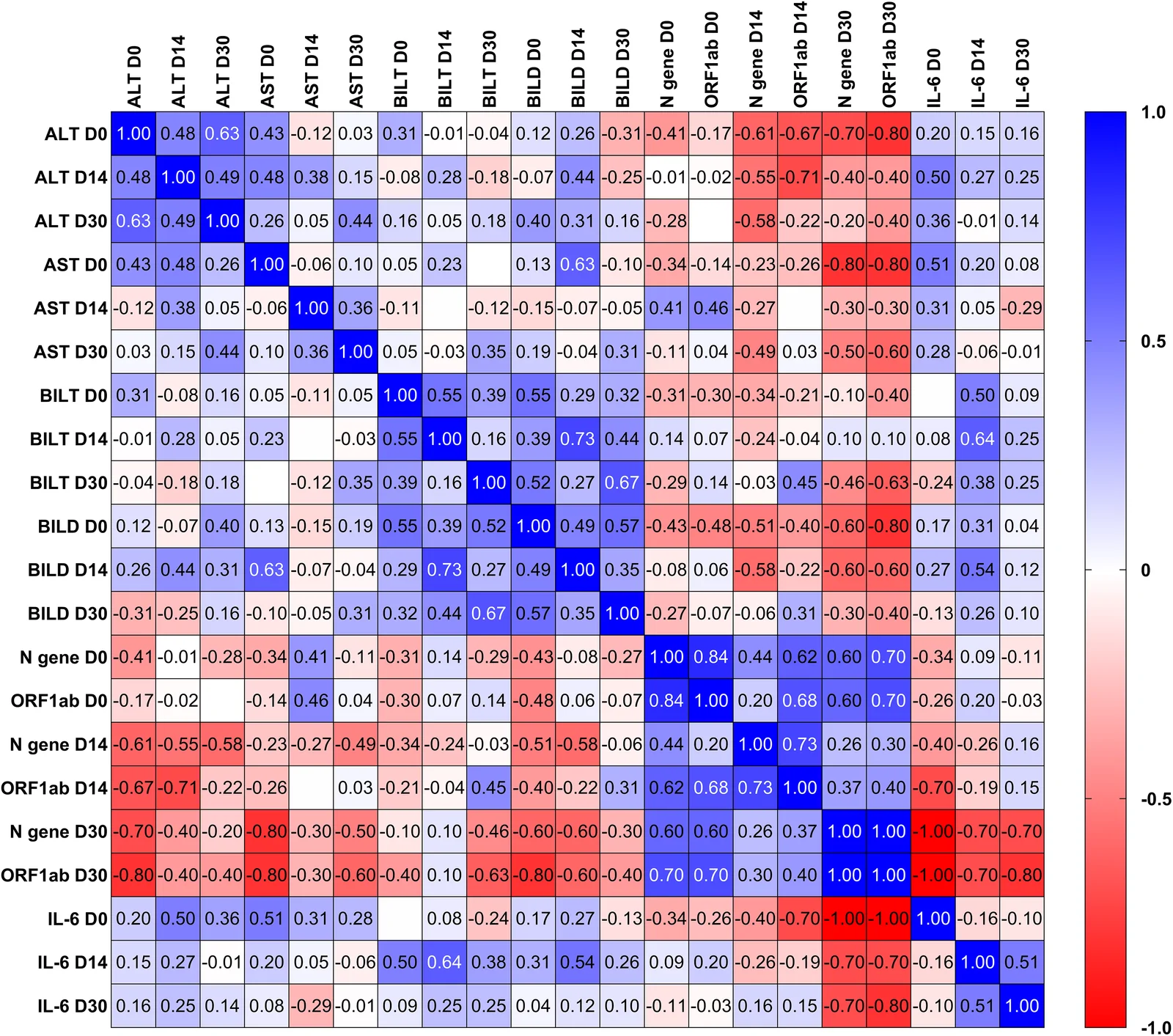
Viral load and inflammatory markers correlated with hepatic biochemical parameters during follow-up. The heat map displays the correlation coefficients (r) for all variables on days 0 (D0), 14 (D14) and 30 (D30) (Fig 7). The colour scale indicates the strength and direction of the association, ranging from red (perfect negative correlation, r = −1.0) to blue (perfect positive correlation, r = 1.0). The variables include transaminases (ALT and AST), bilirubin (BILT and BILD), viral load amplification cycles (N and ORF1ab genes), and interleukin-6 (IL-6).

Spearman’s correlation analysis revealed significant associations between viral load, inflammatory markers, and hepatic biochemical parameters during follow-up. The N gene at D14 showed an inverse relationship with direct bilirubin (BILD) at D14 (r = −0.58, p = 0.032). The ORF1ab gene at D14 showed highly significant negative correlations with ALT at D0 (r = −0.67, p = 0.004) and D14 (r = −0.71, p = 0.006), as well as with return to IL-6 at D0 (r = −0.70, p = 0.006). A moderately significant trend was also observed between N gene viral load at D14 and ALT at D0 (r = −0.61, p = 0.011) and at D14 (r = −0.55, p = 0.043). A non-significant trend was additionally observed between N gene viral load at D14 and ALT at D30 (r = −0.58, p = 0.052). IL-6 at D0 was positively correlated with AST at D0 (r = 0.51, p = 0.007) and with ALT at D14 (r = 0.50, p = 0.024). Lastly, IL-6 at D14 showed positive associations with BILT (r = 0.64, p = 0.001) and BILD (r = 0.54, p = 0.006) on the same day.

## Discussion

In this longitudinal prospective study conducted in Yaoundé, Cameroon, we characterised the temporal dynamics of COVID-19-associated liver injury across three time points, diagnosis(D0), day 14 (D14), and day 30 (D30), in relation to systemic inflammation (IL6), delayed viral clearance, and IgG serostatus. Our findings support a biphasic model of hepatic injury: an early inflammation-driven phase peaking at D14, followed by a post-acute phase in which delayed viral clearance emerges as the principal driver of residual hepatic abnormalities.

The transient peak in ALT and AST at D14, followed by partial normalisation at D30, is consistent with the well-established pattern of acute hepatocellular injury during COVID-19, as reported in large multi-centre cohorts [27,28]. However, our study extends this observation by directly linking the timing of transaminase elevation to concurrent IL-6 dynamics, a pairing that has been reported in severe COVID-19 patients but has been less systematically examined in prospective African cohorts [1,6]. The moderate positive correlation between IL-6 and total bilirubin (BILT) at D14 (r = 0.64; p = 0.001) is of particular biological significance. IL-6 has been shown to downregulate key hepatobiliary transporters, including the bile salt export pump (BSEP) and multidrug resistance-associated protein 2 (MRP2), thereby impairing bilirubin excretion and inducing functional inflammatory cholestasis [29]. This mechanism is distinct from hepatocellular necrosis and may be entirely reversible once systemic inflammation subsides, a process that appears to have occurred in our cohort by D30 in most participants. Comparable patterns have been described in an Asian hospitalised cohort [1,10], but our findings suggest that even in a largely mild-to-moderate African cohort, this inflammatory hepatobiliary signature is reproduced, reinforcing the transdiagnostic relevance of IL-6 and bilirubin. Our results contrast with those of McConnell et al. [10], who documented IL-6-mediated liver endotheliopathy predominantly in severe hospitalised patients. The fact that we observed similar IL-6/bilirubin correlations in a milder, outpatient-dominated cohort suggests that the threshold of inflammatory signalling needed to perturb hepatobiliary transport may be lower than previously appreciated, at least in the biological context of Sub-Saharan African populations where baseline cytokine profiles and genetic polymorphisms of transporters may differ [15,16].

A key finding of our study is the dissociation between IgG seroconversion and normalisation of liver biomarkers. Although IgG seropositivity increased progressively between D14 and D30 (from 14/17–15/17 in the seropositive group), biliary markers (BILT and BILD) showed no corresponding decline during the same period. This decoupling is consistent with accumulating evidence demonstrating that the mere presence of anti-SARS-CoV-2 IgG does not indicate effective viral neutralisation or complete tissue resolution [30,31]. The qualitative characteristics of the humoral response, including antibody avidity, neutralising capacity, and Fc-effector functions, appear to be more determinant of clinical recovery than antibody titres alone [31]. Our data therefore challenge the clinical reflex of equating IgG seroconversion with biological recovery and suggest that biochemical hepatic monitoring should be maintained beyond the point of seroconversion, particularly in resource-limited outpatient settings where liver function tests are not routinely repeated after initial diagnosis. This finding is in line with Seow et al. [30], who demonstrated that IgG kinetics in COVID-19 are highly variable and do not uniformly reflect viral clearance trajectories. Our results add a hepatic dimension to this observation: the persistence of biliary markers in seropositive individuals implies that the liver may continue to experience immune-mediated or viral insults even after adaptive immunity is detectably engaged.

The most original contribution of this study is the demonstration that SARS-CoV-2 RNA persistence beyond D14 is independently associated with elevated ALT and BILT at D30, when systemic IL-6 levels have substantially declined in most participants. The paradoxically lower AST levels at D14 in participants with persistent viral RNA, compared with those who had cleared the virus (p = 0.004), is an unexpected finding that warrants careful interpretation. One plausible explanation is that participants who rapidly cleared the virus between D0 and D14 mounted a more intense early cytolytic hepatic immune response, characterised by peak NK cell and cytotoxic T-lymphocyte activity, resulting in greater hepatocellular damage and AST release at D14 [10,32]. In contrast, participants with delayed clearance may have developed a more attenuated, tolerance-prone immune response that avoids peak transaminase release while sustaining subthreshold hepatic stress over a longer period. This phenotype of immune tolerance or exhaustion has been described in viral infections and during SARS-CoV-2 infection, where T-cell and NK-cell exhaustion limits acute immunopathology at the expense of delayed viral clearance [33–35]. However, this interpretation is speculative: the small group sizes limit statistical confidence, and we cannot rule out that this finding reflects sampling variability. This result also helps explain why the multivariable mixed-effect models do not show a significant overall association between Ct N gene and AST after full adjustment, as the relationship between viral RNA burden and transaminase levels is non-linear and intricately mediated by host immune response kinetics [6,35]. The strong negative correlations between Ct values for both N and ORF1ab genes at D14 and liver biomarkers at D0 and D14 indicate that higher viral replication is temporally linked to greater hepatocellular stress. This pattern supports the hypothesis that the liver acts as a transient viral reservoir, as previously proposed based on high ACE2 expression on cholangiocytes and, to a lesser extent, hepatocytes [1,6,36]. Persistent viral material within hepatic tissue may sustain Kupffer cell activation and local innate immune signalling, thereby maintaining focal hepatic injury independently of circulating cytokine concentrations [16]. This concept of a smoldering hepatic viral reservoir has been proposed as one of the mechanistic pillars of long COVID-associated liver dysfunction, a syndrome characterised by persistent or recurrent hepatic enzyme abnormalities weeks to months after acute infection [37,38]. Our prospective data, obtained from a defined Cameroonian cohort, provide direct longitudinal evidence for this hypothesis at the 30-day follow-up.

Importantly, these findings diverge from studies conducted in Asian cohorts that reported rapid and complete normalisation of liver enzymes within 30 days of diagnosis in mild COVID-19 [36,37]. The difference may reflect biological heterogeneity in hepatic viral tropism, immune-resolution kinetics, or the confounding influence of comorbid conditions, including subclinical malaria burden, hepatitis B carrier status, or traditional herbal medicine use, that were controlled by exclusion criteria in our study but may nonetheless differentially shape recovery trajectories in this population.

To disentangle the independent contributions of the biological axes described above, we constructed multivariable linear mixed-effects models for the four primary liver biomarker outcomes across all three time points. These analyses yield three findings that substantively extend and confirm the univariate observations. First, IL-6 was the sole significant independent predictor of AST after adjustment for age, sex, symptom status, viral load, and IgG serostatus, confirming that systemic inflammation is a genuine driver of hepatocellular injury rather than merely a correlate of disease severity. This is consistent with the well-established concept of bystander hepatitis, whereby systemic hypercytokinaemia drives hepatocellular injury through indirect inflammatory mechanisms [39–42]. At the molecular level, IL-6 trans-signalling via gp130 on liver sinusoidal endothelial cells triggers NF-κB activation and ROS production, thereby amplifying oxidative stress in hepatocytes and promoting mitochondrial dysfunction. The selective association of IL-6 with AST rather than ALT may reflect preferential centrilobular injury consistent with sinusoidal endotheliopathy, which disproportionately affects zone 3 hepatocytes, the predominant source of mitochondrial AST release [10,43,44]. Second, viral RNA burden (Ct N gene) was an independent predictor of direct bilirubin after full adjustment for Il-6, pointing to a parallel, inflammation-independent cholestatic pathway. The most plausible mechanism is direct SARS-CoV-2 cytopathic injury to ACE2-expressing cholangiocytes, which downregulates canalicular transporters and impairs bile secretion independently of the IL-driven BSEP/MRP2 suppression pathway active at the hepatocyte level [45,46]. The independent coexistence of both mechanisms in our models supports a dual-pathway model of COVID-19-associated cholestasis: an early inflammatory phase driven by IL-6-mediated transporter suppression and a sustained direct biliary phase driven by cholangiocyte injury proportional to viral RNA burden, the latter persisting as systemic inflammation subsides [47]. Third, IgG seropositivity was paradoxically and independently associated with higher BILD after adjustment for viral load and IL-6, reinforcing our central conclusion that qualitative seroconversion does not mark hepatobiliary resolution. This counterintuitive finding may reflect that IgG positivity in this context identifies individuals with greater cumulative antigen exposure and, correspondingly, greater cholangiocyte-mediated biliary stress, rather than indicating effective viral clearance [30]. Alternatively, immune complex formation and complement activation via the classical pathway, triggered by circulating IgG-antigen complexes, may amplify local biliary injury independently of IL-6 [37,48,49]. Persistent elevation of biliary markers despite IgG seropositivity has similarly been observed in post-COVID longitudinal follow-up data, confirming that hepatic perturbation is not solely determined by viral load magnitude or immune response detection [15,50]. Finally, the consistently high conditional R² values for ALT (0.798) and BILT (0.853), relative to their low marginal R² values (0.190 and 0.170), indicate that most longitudinal variance in these biomarkers is attributable to stable between-subject heterogeneity captured by individual random intercepts, rather than to the time-varying fixed predictors measured here. This likely reflects unmeasured individual-level variables prevalent in sub-Saharan African populations, including subclinical NAFLD, traditional medication, and cumulative environmental exposures, which are recognised as drivers of inter-individual variability in liver enzyme responses to infectious triggers [50]. From a Long COVID perspective, the incomplete hepatic resolution observed at D30 in our cohort aligns with the broader literature documenting persistent liver function abnormalities as part of the post-acute sequelae of SARS-CoV-2 infection, necessitating prolonged monitoring beyond the acute phase [15,51].

Participants aged >30 years exhibited significantly higher direct bilirubin levels at D14 and D30 than their younger counterparts, along with persistently elevated IL-6 concentrations at D14. While 30 years would not typically be considered a clinically meaningful age threshold in Western hepatology, it may represent a relevant biological inflexion point in the Cameroonian demographic context, where cumulative environmental exposures, endemic infection burden, and accelerated biological ageing may shift liver function trajectories earlier than in low-endemicity settings [52]. This pattern is reminiscent of inflammaging, the chronic, low-grade inflammatory state associated with advancing age, which is characterised by impaired resolution of cytokine peaks and reduced efficiency of canalicular transport systems [52,53]. Age-related endothelial dysfunction further impairs intrahepatic microcirculation, potentially prolonging cholestatic injury following inflammatory insults [53]. These findings invite caution when applying age-adjusted reference ranges derived from Western cohorts to Sub-Saharan African populations and suggest that post-COVID hepatic monitoring may need to be extended preferentially in patients over 30 in this epidemiological context.

Male participants showed significantly higher IL-6 levels at D0, consistent with the well-documented sexual dimorphism in innate immune cytokine responses and the higher burden of severe COVID-19 in men reported globally [54,55]. Interestingly, this difference was attenuated at D14 and D30, suggesting that although male sex is associated with a more intense initial inflammatory response, resolution kinetics did not differ substantially between the sexes in our cohort. Greater variability in transaminase levels at D14 among men may reflect the combined effect of this early inflammatory peak and potential sex-specific differences in ACE2 expression and drug-metabolising enzyme activity, though our study was not powered to test these mechanisms directly.

As expected, symptomatic patients showed significantly higher IL-6 levels at D0 than asymptomatic individuals, confirming the role of early cytokine surges in shaping clinical manifestations [54]. The apparent reversal at D30, where asymptomatic individuals exhibited higher IL-6 levels than symptomatic individuals, is a counterintuitive but biologically plausible finding. It may reflect sustained low-grade immune activation in the absence of clinically manifest disease, a phenomenon observed in other viral infections, where immune resolution proceeds differently when the initial pathogen dose or inflammatory surge is below the symptomatic threshold [56]. Alternatively, this reversal could be a statistical artefact arising from the reduced sample sizes at D30 and warrants careful interpretation. Overall, these findings support a biphasic model of COVID-19–related liver injury, characterised by an early inflammation-driven stage followed by a post-acute phase where viral persistence largely delays hepatic recovery.

Several limitations of this study should be acknowledged. First, the sample size (n = 38) is modest and may limit the statistical power to detect associations across subgroups, particularly when stratified by sex, age, or symptom status. Larger prospective cohorts are needed to confirm and extend these findings. Second, the 30-day follow-up after diagnosis may be insufficient to fully understand the course of hepatic recovery in patients with viral persistence. Extending the follow-up to 90 days or beyond would be particularly valuable given the emerging evidence on Long COVID-associated hepatic sequelae. Third, because patients with known hepatitis B or C co-infection were excluded to avoid confounding, our findings may not be generalisable to the broader Cameroonian population, in which these co-infections are highly prevalent and may fundamentally alter the host’s response to SARS-CoV-2. Fourth, liver injury was assessed exclusively through serum biomarkers; liver biopsies or imaging assessments (e.g., elastography) were unavailable, precluding characterisation of the structural hepatic changes underlying the observed biochemical alterations. Five, we cannot fully exclude the contribution of drug-induced liver injury, as some participants may have received hepatotoxic medicines, including traditional herbal remedies, during the follow-up period, although participants were queried about medication use at each visit. Sixth, the absence of a local healthy control group and the lack of pre-infection biomarker measurements mean that D0 values cannot be validated as a healthy baseline. All longitudinal comparisons should be interpreted in the context of participants already infected at D0. Finally, IgG and IgM serological status was assessed using rapid immunochromatographic tests, which do not provide quantitative antibody titres, limiting the granularity of humoral immune analysis. Future studies incorporating quantitative IgG assays, avidity measurements, and assessments of neutralisation capacity would enable a more refined characterisation of the relationship between humoral immunity and hepatic recovery. Despite these shortcomings, this study remains, to our knowledge, among the first prospective longitudinal characterisations of COVID-19-associated hepatic kinetics in a Sub-Saharan African cohort, addressing a critical evidence gap in a population where baseline liver physiology and viral-host dynamics may differ from those reported in resource-privilege settings. The reliance on serum biomarkers rather than biopsy or imaging, while precluding structural insight, reflects the pragmatic standard of care in this resource-limited context and enhances the direct applicability of our findings to similar healthcare settings where advanced hepatological tools are largely unavailable.

## Conclusion

This prospective longitudinal study, conducted in Yaoundé, Cameroon, provides evidence that COVID-19-associated liver injury follows a biphasic course in a Sub-Saharan African outpatient cohort. During the acute phase (D0-D14), hepatic alteration was characterised by transient transaminase elevation and cholestatic bilirubin disturbances, are primarily driven by systemic IL-6-mediated inflammation, rather than direct viral cytopathic effect alone. In the post-acute phase (D14-D30), as systemic inflammation subsides, residual viral RNA persistence becomes the principal determinant of ongoing hepatic abnormalities, independent of IgG seroconversion status. Furthermore, participants aged >30 years demonstrate greater vulnerability to prolonged biliary perturbations, suggesting that age-related shifts in immune resolution and hepatobiliary transport efficiency are relevant even within relatively young African cohorts.

These findings suggest clinical and public health implications. They challenge the common clinical assumption that IgG seroconversion marks the end of biologically relevant disease activity, and instead underscore the need for extended biochemical monitoring of liver function beyond the acute phase, particularly in patients with delayed viral clearance, symptomatic presentation, or age > 30 years. In resource-limited healthcare settings such as those prevalent across Sub-Saharan Africa, where post-acute follow-up is frequently curtailed by infrastructure and economic constraints, these data argue for integrating post-COVID hepatic surveillance into existing infectious disease management protocols.

From a translational perspective, the observed IL-6/bilirubin axis and the association between delayed viral clearance and residual liver injury suggest two potentially actionable therapeutic targets. Anti-IL-6 or IL-6 receptor antagonist strategies, already deployed in severe COVID-19, may warrant evaluation in moderate cases with early hepatobiliary involvement. Concurrently, strategies to accelerate viral clearance, including antiviral therapy during the post-acute window, could reduce the duration of liver stress in patients with documented RNA persistence beyond D14.

More broadly, this study positions the liver as a clinically informative organ for tracking systemic viral burden and immune resolution dynamics in COVID-19. The hepatic biomarker trajectory described here may serve as a practical, non-invasive surrogate for viral reservoir activity in ambulatory patients and may be relevant to long COVID monitoring in settings where advanced imaging or virological follow-up is not feasible. Future studies should extend follow-up beyond 30 days, incorporate histopathological or elastographic assessment of hepatic injury, and include populations with hepatitis B, HIV, or malaria co-infection to better reflect the full epidemiological complexity of COVID-19 recovery in Sub-Saharan Africa.

## Supporting information

S1 Table Sociodemographic and longitudinal clinical/biomarker characteristics. (DOCX)

S1 File Deidentified database. (XLSX)

## References

[1] Zhang C, Shi L, Wang F-S. Liver injury in COVID-19: Management and challenges. Lancet Gastroenterol Hepatol. 2020;5(5):428–30. doi: 10.1016/S2468-1253(20)30057-1 32145190 PMC7129165

[2] Nardo AD, Schneeweiss-Gleixner M, Bakail M, Dixon ED, Lax SF, Trauner M. Pathophysiological mechanisms of liver injury in COVID-19. Liver Int. 2021;41(1):20–32. doi: 10.1111/liv.14730 33190346 PMC7753756

[3] Fan Z, Chen L, Li J, Cheng X, Yang J, Tian C, et al. Clinical features of COVID-19-related liver functional abnormality. Clin Gastroenterol Hepatol. 2020;18(7):1561–6. doi: 10.1016/j.cgh.2020.04.002 32283325 PMC7194865

[4] Cai Q, Huang D, Yu H, Zhu Z, Xia Z, Su Y, et al. COVID-19: Abnormal liver function tests. J Hepatol. 2020;73(3):566–74. doi: 10.1016/j.jhep.2020.04.006 PMC7194951 32298767

[5] Shi Y, Wang M, Wu L, Li X, Liao Z. COVID-19 associated liver injury: An updated review on the mechanisms and management of risk groups. Liver Res. 2023;7(3):207–15. doi: 10.1016/j.livres.2023.07.001 39958382 PMC11792068

[6] Cai Y, Ye L-P, Song Y-Q, Mao X-L, Wang L, Jiang Y-Z, et al. Liver injury in COVID-19: Detection, pathogenesis, and treatment. World J Gastroenterol. 2021;27(22):3022–36. doi: 10.3748/wjg.v27.i22.3022 34168405 PMC8192279

[7] Taneva G, Dimitrov D, Velikova T. Liver dysfunction as a cytokine storm manifestation and prognostic factor for severe COVID-19. World J Hepatol. 2021;13(12):2005–12. doi: 10.4254/wjh.v13.i12.2005 35070004 PMC8727206

[8] Liu T, Zhang J, Yang Y, Ma H, Li Z, Zhang J, et al. The role of interleukin-6 in monitoring severe case of coronavirus disease 2019. EMBO Mol Med. 2020;12(7):e12421. doi: 10.15252/emmm.202012421 32428990 PMC7280589

[9] Broman N, Rantasärkkä K, Feuth T, Valtonen M, Waris M, Hohenthal U, et al. IL-6 and other biomarkers as predictors of severity in COVID-19. Ann Med. 2021;53(1):410–2. doi: 10.1080/07853890.2020.1840621 33305624 PMC7935117

[10] McConnell MJ, Kawaguchi N, Kondo R, Sonzogni A, Licini L, Valle C, et al. Liver injury in COVID-19 and IL-6 trans-signaling-induced endotheliopathy. J Hepatol. 2021;75(3):647–58. doi: 10.1016/j.jhep.2021.04.050 33991637 PMC8285256

[11] Dhar SK, K V, Damodar S, Gujar S, Das M. IL-6 and IL-10 as predictors of disease severity in COVID-19 patients: Results from meta-analysis and regression. Heliyon. 2021;7(2):e06155. doi: 10.1016/j.heliyon.2021.e06155 33553782 PMC7846230

[12] Del Zompo F, De Siena M, Ianiro G, Gasbarrini A, Pompili M, Ponziani FR. Prevalence of liver injury and correlation with clinical outcomes in patients with COVID-19: Systematic review with meta-analysis. Eur Rev Med Pharmacol Sci. 2020;24(24):13072–88. doi: 10.26355/eurrev_202012_24215 33378061

[13] Fahmi M, Kharisma VD, Ansori ANM, Ito M. Retrieval and investigation of data on SARS-CoV-2 and COVID-19 using bioinformatics approach. In: Rezaei N, éditeur. Coronavirus disease - COVID-19 [Internet]. Cham: Springer International Publishing; 2021 [cité 2026 juill 14]. p. 839–57. Disponible sur: doi: 10.1007/978-3-030-63761-3_47 33973215

[14] Cucunawangsih C, Ansori ANM, Vatvani AD, Hariyanto TI. Impact of nirmatrelvir/ritonavir on the risk of long COVID in outpatients: A systematic review and meta-analysis. Expert Rev Anti Infect Ther. 2026;24(2):271–84. doi: 10.1080/14787210.2026.2636175 41717886

[15] Florea C-E, Bălaș-Maftei B, Rotaru A, Abudanii PL, Vieru ST, Grigoriu M, et al. Multiorgan involvement and particularly liver injury in Long COVID: A narrative review. Life (Basel). 2025;15(8):1314. doi: 10.3390/life15081314 40868961 PMC12387570

[16] Wölfel R, Corman VM, Guggemos W, Seilmaier M, Zange S, Müller MA, et al. Virological assessment of hospitalized patients with COVID-2019. Nature. 2020;581(7809):465–9. doi: 10.1038/s41586-020-2196-x 32235945

[17] Wang Y, Zhang L, Sang L, Ye F, Ruan S, Zhong B, et al. Kinetics of viral load and antibody response in relation to COVID-19 severity. J Clin Invest. 2020;130(10):5235–44. doi: 10.1172/JCI138759 32634129 PMC7524490

[18] Lucas C, Klein J, Sundaram ME, Liu F, Wong P, Silva J, et al. Delayed production of neutralizing antibodies correlates with fatal COVID-19. Nat Med. 2021;27(7):1178–86. doi: 10.1038/s41591-021-01355-0 33953384 PMC8785364

[19] Khoury DS, Cromer D, Reynaldi A, Schlub TE, Wheatley AK, Juno JA, et al. Neutralizing antibody levels are highly predictive of immune protection from symptomatic SARS-CoV-2 infection. Nat Med. 2021;27(7):1205–11. doi: 10.1038/s41591-021-01377-8 34002089

[20] Cissé F, Gueye M, Thiam S, Ndiaye A, Samba A, AgneF D, et al. Transaminase reference values in a Senegalese adult population. 2018;8(8):13–6.

[21] Fiseha T, Alemayehu E, Mohammed Adem O, Eshetu B, Gebreweld A. Reference intervals for common clinical chemistry parameters in healthy adults of Northeast Ethiopia. PLoS One. 2022;17(11):e0276825. doi: 10.1371/journal.pone.0276825 36322541 PMC9629653

[22] Shrestha AB, Aryal M, Magar JR, Shrestha S, Hossainy L, Rimti FH. The scenario of self-medication practices during the covid-19 pandemic; A systematic review. Ann Med Surg (Lond). 2022;82:104482. doi: 10.1016/j.amsu.2022.104482 36059596 PMC9419440

[23] Li P, Liu Y, Cheng Z, Yu X, Li Y. COVID-19-associated liver injury: Clinical characteristics, pathophysiological mechanisms and treatment management. Biomed Pharmacother. 2022;154:113568. doi: 10.1016/j.biopha.2022.113568 36029543 PMC9381432

[24] Jothimani D, Venugopal R, Abedin MF, Kaliamoorthy I, Rela M. COVID-19 and the liver. J Hepatol. 2020;73(5):1231–40. doi: 10.1016/j.jhep.2020.06.006 32553666 PMC7295524

[25] Tene H, Ngamaleu R, Djounda R, Minomo R, Ngale S, Besong M, et al. Cytokines dynamics and biological sex differences in SARS-CoV-2 infected people in Cameroon. Clin Exp Immunol. 2025;219(1):uxaf063. doi: 10.1093/cei/uxaf063 40982477 PMC12687354

[26] Organization WH. Considerations in the investigation of cases and clusters of COVID-19: Interim guidance [Internet]; 2020 [cité 2026 juill 27]; WHO/2019-nCoV/cases_clusters_investigation/2020.2. Disponible sur: https://iris.who.int/handle/10665/331668

[27] Diaz-Louzao C, Barrera-Lopez L, Lopez-Rodriguez M, Casar C, Vazquez-Agra N, Pernas-Pardavila H, et al. Longitudinal relationship of liver injury with inflammation biomarkers in COVID-19 hospitalized patients using a joint modeling approach. Sci Rep. 2022;12(1):5547. doi: 10.1038/s41598-022-09290-x 35365705 PMC8972986

[28] Yang R, Feng J, Wan H, Zeng X, Ji P, Zhang J. Liver injury associated with the severity of COVID-19: A meta-analysis. Front Public Health. 2023;11:1003352. doi: 10.3389/fpubh.2023.1003352 36817905 PMC9932800

[29] Sivandzadeh GR, Askari H, Safarpour AR, Ejtehadi F, Raeis-Abdollahi E, Vaez Lari A, et al. COVID-19 infection and liver injury: Clinical features, biomarkers, potential mechanisms, treatment, and management challenges. World J Clin Cases. 2021;9(22):6178–200. doi: 10.12998/wjcc.v9.i22.6178 34434987 PMC8362548

[30] Seow J, Graham C, Merrick B, Acors S, Pickering S, Steel KJA, et al. Longitudinal evaluation and decline of antibody responses in SARS-CoV-2 infection. Nat Microbiol. 2020;5(12):1598–607. doi: 10.1038/s41564-020-00813-8 33106674 PMC7610833

[31] Lapuente D, Winkler TH, Tenbusch M. B-cell and antibody responses to SARS-CoV-2: Infection, vaccination, and hybrid immunity. Cell Mol Immunol. 2024;21(2):144–58. doi: 10.1038/s41423-023-01095-w 37945737 PMC10805925

[32] Iannacone M, Guidotti LG. Immunobiology and pathogenesis of hepatitis B virus infection. Nat Rev Immunol. 2022;22(1):19–32. doi: 10.1038/s41577-021-00549-4 34002067

[33] Zheng M, Gao Y, Wang G, Song G, Liu S, Sun D, et al. Functional exhaustion of antiviral lymphocytes in COVID-19 patients. Cell Mol Immunol. 2020;17(5):533–5. doi: 10.1038/s41423-020-0402-2 32203188 PMC7091858

[34] Wherry EJ. T cell exhaustion. Nat Immunol. 2011;12(6):492–9. doi: 10.1038/ni.2035 21739672

[35] Lucas C, Wong P, Klein J, Castro TBR, Silva J, Sundaram M, et al. Longitudinal analyses reveal immunological misfiring in severe COVID-19. Nature. 2020;584(7821):463–9. doi: 10.1038/s41586-020-2588-y 32717743 PMC7477538

[36] Roshanshad R, Roshanshad A, Fereidooni R, Hosseini-Bensenjan M. COVID-19 and liver injury: Pathophysiology, risk factors, outcome and management in special populations. World J Hepatol. 2023;15(4):441–59. doi: 10.4254/wjh.v15.i4.441 37206656 PMC10190688

[37] Sherif ZA, Gomez CR, Connors TJ, Henrich TJ, Reeves WB, RECOVER Mechanistic Pathway Task Force. Pathogenic mechanisms of post-acute sequelae of SARS-CoV-2 infection (PASC). Rosen CJ, Zaidi M, éditeurs. eLife. 2023;12:e86002. doi: 10.7554/eLife.86002 PMC10032659 36947108

[38] Chen B, Julg B, Mohandas S, Bradfute SB, RECOVER Mechanistic Pathways Task Force. Viral persistence, reactivation, and mechanisms of long COVID. Iqbal J, Zaidi M, éditeurs. eLife. 2023;12:e86015. doi: 10.7554/eLife.86015 PMC10159620 37140960

[39] Wang Q, Zhao H, Liu L-G, Wang Y-B, Zhang T, Li M-H, et al. Pattern of liver injury in adult patients with COVID-19: A retrospective analysis of 105 patients. Mil Med Res. 2020;7(1):28. doi: 10.1186/s40779-020-00256-6 32507110 PMC7276224

[40] Liao X, Li D, Ma Z, Zhang L, Zheng B, Li Z. 12-month post-discharge liver function test abnormalities among patients with COVID-19: A single-center prospective cohort study. Front Cell Infect Microbiol. 2022;12. doi: 10.3389/fcimb.2022.864933 PMC9046934 35493732

[41] Diamond T, Bennett AD, Behrens EM. The liver in hemophagocytic lymphohistiocytosis: Not an innocent bystander. J Pediatr Gastroenterol Nutr. 2023;77(2):153–9. doi: 10.1097/MPG.0000000000003807 37098099 PMC10524294

[42] Soy M, Atagündüz P, Atagündüz I, Sucak GT. Hemophagocytic lymphohistiocytosis: A review inspired by the COVID-19 pandemic. Rheumatol Int. 2021;41(1):7–18. doi: 10.1007/s00296-020-04636-y 32588191 PMC7315691

[43] Weidinger A, Dungel P, Perlinger M, Singer K, Ghebes C, Duvigneau JC, et al. Experimental data suggesting that inflammation mediated rat liver mitochondrial dysfunction results from secondary hypoxia rather than from direct effects of inflammatory mediators. Front Physiol. 2013;4:138. doi: 10.3389/fphys.2013.00138 23760194 PMC3675332

[44] Yao R, Xu G, Fu X, Zhang W, Wang H, Chen Y, et al. Clinical characteristics and the role of IL-6 in acute-on-chronic liver failure patients with or without COVID-19: A multicenter paired cohort study. Front Cell Infect Microbiol. 2025;14:1471974. doi: 10.3389/fcimb.2024.1471974 39867341 PMC11757239

[45] Zhao B, Ni C, Gao R, Wang Y, Yang L, Wei J, et al. Recapitulation of SARS-CoV-2 infection and cholangiocyte damage with human liver ductal organoids. Protein Cell. 2020;11(10):771–5. doi: 10.1007/s13238-020-00718-6 32303993 PMC7164704

[46] Chai X, Hu L, Zhang Y, Han W, Lu Z, Ke A, et al. Specific ACE2 expression in cholangiocytes may cause liver damage after 2019-nCoV infection [Internet]. bioRxiv; 2020 [cité 2026 juill 29]. 2020.02.03.931766 p. Disponible sur: https://www.biorxiv.org/content/10.1101/2020.02.03.931766v1

[47] Herta T, Berg T. COVID-19 and the liver - Lessons learned. Liver Int. 2021;41 Suppl 1(Suppl 1):1–8. doi: 10.1111/liv.14854 34155789 PMC8447354

[48] Cargill T, Barnes E, Rispens T, Culver EL. The differential complement, Fc and chemokine receptor expression of B cells in IgG4-related pancreatobiliary disease and primary sclerosing cholangitis and its relevance for targeting B cell pathways in disease. Biomedicines. 2024;12(12):2839. doi: 10.3390/biomedicines12122839 39767745 PMC11673969

[49] Naeem M, Bano N, Manzoor S, Ahmad A, Munawar N, Razak SIA, et al. Pathogenetic mechanisms of liver-associated injuries, management, and current challenges in COVID-19 patients. Biomolecules. 2023;13(1):99. doi: 10.3390/biom13010099 36671484 PMC9855873

[50] Lebbe A, Aboulwafa A, Bayraktar N, Mushannen B, Ayoub S, Sarker S, et al. New onset of acute and chronic hepatic diseases post-COVID-19 infection: A systematic review. Biomedicines. 2024;12(9):2065. doi: 10.3390/biomedicines12092065 39335578 PMC11428502

[51] Stasi C. Post-COVID-19 pandemic sequelae in liver diseases. Life (Basel). 2025;15(3):403. doi: 10.3390/life15030403 40141748 PMC11943493

[52] Hossain S, Islam S, Kamruzzaman M, Shekhar HU. Exploring age-related influences on COVID-19 severity and therapeutic approaches for older patients. Aging Health Res. 2025;5(3):100259. doi: 10.1016/j.ahr.2025.100259

[53] Zhao S-W, Li Y-M, Li Y-L, Su C. Liver injury in COVID-19: Clinical features, potential mechanisms, risk factors and clinical treatments. World J Gastroenterol. 2023;29(2):241–56. doi: 10.3748/wjg.v29.i2.241 36687127 PMC9846943

[54] Chen X, Zhao B, Qu Y, Chen Y, Xiong J, Feng Y, et al. Detectable serum SARS-CoV-2 viral load (RNAaemia) is closely correlated with drastically elevated interleukin 6 (IL-6) level in critically ill COVID-19 patients. Clin Infect Dis. 2020. doi: 10.1093/cid/ciaa449 32301997 PMC7184354

[55] Huang C, Wang Y, Li X, Ren L, Zhao J, Hu Y, et al. Clinical features of patients infected with 2019 novel coronavirus in Wuhan, China. Lancet. 2020;395(10223):497–506. doi: 10.1016/S0140-6736(20)30183-5 31986264 PMC7159299

[56] Gao Y, Li T, Han M, Li X, Wu D, Xu Y, et al. Diagnostic utility of clinical laboratory data determinations for patients with the severe COVID-19. J Med Virol. 2020;92(7):791–6. doi: 10.1002/jmv.25770 32181911 PMC7228247

