# Supplementary material for: Early inflammation-driven damage and delayed viral clearance are associated with prolonged biliary perturbation in a Cameroonian cohort of COVID-19 patients
Source: PLoS One. 2026 Oct 16;21(10):e0357503. doi: 10.1371/journal.pone.0357503 (PMC13674779; doi:10.1371/journal.pone.0357503)
Supplement: Supplementary file 1 [file pone.0357503.s001.docx]

**Supplementary Table 1. Sociodemographic and Longitudinal Clinical/Biomarker Characteristics**

| **Characteristic** | **Overall (N=38)** | **Symptomatic (n=17)** | **Asymptomatic (n=21)** | **Difference (95% CI)** | **Effect Size** | **p-value (FDR adj.)** |
| --- | --- | --- | --- | --- | --- | --- |
| **Demographics** | | | | | | |
| Male Sex, n (%) | 19 (50.0%) | 9 (52.9%) | 10 (47.6%) | 5.3% (-31.9 to 42.6) | OR = 1.23 | 1.000 (1.000) |
| Age (years), Median (IQR) | 31.0 (24.2–45.0) | 33.0 (25.0–49.0) | 31.0 (24.0–35.0) | 3.00 (-6.00 to 15.00) | r = 0.12 (negligible) | 0.537 (0.754) |
| **Transaminases (IU/L)** | | | | | | |
| ALT — Day 0 | 17.6 (14.1–24.4) | 17.4 (11.9–23.8) | 17.7 (15.3–24.0) | -1.50 (-7.50 to 5.00) | r = -0.11 (negligible) | 0.621 (0.754) |
| ALT — Day 14 | 17.5 (13.9–24.2) | 18.2 (14.8–24.4) | 17.1 (13.4–23.9) | 0.86 (-6.10 to 7.40) | r = 0.08 (negligible) | 0.752 (0.853) |
| ALT — Day 30 | 16.5 (13.0–21.9) | 16.8 (14.9–22.9) | 16.1 (9.7–21.1) | 4.14 (-3.10 to 9.40) | r = 0.31 (small) | 0.208 (0.754) |
| AST — Day 0 | 29.6 (22.8–41.2) | 29.9 (24.9–54.0) | 29.4 (21.1–36.4) | 4.50 (-5.00 to 19.30) | r = 0.23 (small) | 0.312 (0.754) |
| AST — Day 14 | 36.4 (27.0–64.5) | 44.3 (34.1–62.2) | 35.3 (24.1–65.7) | 6.80 (-11.10 to 21.90) | r = 0.16 (small) | 0.488 (0.754) |
| AST — Day 30 | 35.1 (28.4–42.6) | 35.7 (30.5–42.5) | 31.7 (26.9–41.8) | 2.95 (-7.40 to 13.50) | r = 0.12 (negligible) | 0.616 (0.754) |
| **Bilirubin (mg/dL)** | | | | | | |
| Total Bilirubin — Day 0 | 0.3 (0.2–0.4) | 0.3 (0.2–0.3) | 0.3 (0.3–0.4) | -0.05 (-0.15 to 0.05) | r = -0.26 (small) | 0.252 (0.754) |
| Total Bilirubin — Day 14 | 0.4 (0.3–0.5) | 0.3 (0.2–0.4) | 0.5 (0.3–0.5) | -0.12 (-0.25 to -0.02) | r = -0.48 (large) | 0.034 (0.195) |
| Total Bilirubin — Day 30 | 0.3 (0.2–0.5) | 0.3 (0.2–0.5) | 0.4 (0.3–0.5) | -0.07 (-0.21 to 0.09) | r = -0.20 (small) | 0.410 (0.754) |
| Direct Bilirubin — Day 0 | 0.1 (0.1–0.2) | 0.1 (0.1–0.2) | 0.1 (0.1–0.2) | 0.00 (-0.05 to 0.05) | r = 0.01 (negligible) | 0.966 (1.000) |
| Direct Bilirubin — Day 14 | 0.2 (0.1–0.2) | 0.2 (0.1–0.2) | 0.2 (0.1–0.3) | -0.03 (-0.09 to 0.05) | r = -0.19 (small) | 0.409 (0.754) |
| Direct Bilirubin — Day 30 | 0.2 (0.1–0.2) | 0.1 (0.1–0.2) | 0.2 (0.2–0.2) | -0.03 (-0.09 to 0.05) | r = -0.20 (small) | 0.410 (0.754) |
| **Interleukin-6 / IL-6 (pg/mL)** | | | | | | |
| IL-6 — Day 0 | 2.5 (1.3–4.1) | 3.8 (2.6–6.5) | 1.9 (1.2–3.0) | 1.76 (0.36 to 4.16) | r = 0.55 (large) | 0.019 (0.195) |
| IL-6 — Day 14 | 2.5 (1.2–4.5) | 2.8 (1.1–3.7) | 2.5 (1.6–4.7) | -0.64 (-2.51 to 1.34) | r = -0.17 (small) | 0.479 (0.754) |
| IL-6 — Day 30 | 1.6 (0.8–3.2) | 1.1 (0.6–1.7) | 3.2 (1.7–7.3) | -1.84 (-6.03 to -0.22) | r = -0.56 (large) | 0.030 (0.195) |

***Abbreviations:*** *IQR, interquartile range; OR, odds ratio; CI, confidence interval; ALT, alanine aminotransferase; AST, aspartate aminotransferase; IL-6, interleukin-6.*

***Effect sizes:*** *rank-biserial correlation (r) for continuous variables (Mann–Whitney U test); odds ratio (OR) for categorical variables (Fisher's exact test).*

***p-values:*** *raw (Mann–Whitney U or Fisher's exact test) and FDR-adjusted (Benjamini–Hochberg). * q < 0.05 after FDR correction.*

In this cohort of 38 participants, symptomatic and asymptomatic individuals had broadly similar demographic and liver function profiles, with negligible differences in sex distribution, age, ALT, AST, and direct bilirubin across time points. Notable exploratory trends emerged in inflammatory and bilirubin markers: symptomatic participants had higher IL-6 at baseline (Day 0), whereas asymptomatic participants showed elevated IL-6 at Day 30. Both showed large effect sizes but were not significant after correction. Additionally, total bilirubin was lower in symptomatic individuals at Day 14 than in asymptomatic peers. Overall, although no differences remained statistically significant after FDR adjustment, these patterns suggest dynamic shifts in IL-6 and bilirubin that may distinguish symptomatic from asymptomatic trajectories.
